# Exosomes and cardioprotection – A critical analysis

**DOI:** 10.1016/j.mam.2017.11.004

**Published:** 2018-04

**Authors:** Sean M. Davidson, Derek M. Yellon

**Affiliations:** The Hatter Cardiovascular Institute, University College London, 67 Chenies Mews, WC1E 6HX London, United Kingdom

**Keywords:** Exosomes, Cardioprotection, Ischaemia, Reperfusion, Stem cells, Heart

## Abstract

Exosomes are nano-sized vesicles released by numerous cell types that appear to have diverse beneficial effects on the injured heart. Studies using exosomes from stem cells or from the blood have indicated that they are able to protect the heart both in models of acute ischaemia and reperfusion, and during chronic ischaemia. In addition to decreasing initial infarct size, they are able to stimulate angiogenesis, reduce fibrosis and remodelling, alter immune cell function and improve long-term cardiac contractile function. However, since the technology and techniques used for the study of exosomes is relatively immature and continually evolving, there remain many important caveats to the interpretation of studies. This review presents a critical analysis of the field of exosomes and cardioprotection. We analyse the effects of exosomes from all types of stem cells investigated to date, summarize the major effects observed and their potential mechanism, and offer our perspective on the major outstanding issues.

## 1 Introduction

The involvement of larger extracellular vesicles (EVs) such as microparticles or microvesicles (MVs) in thrombosis has been understood for many years (Boulanger et al., 2017). MVs are also understood to be a potential source of biomarkers of pathology (Jansen et al., 2017, Sluijter et al., 2017). More recently, interest has turned to their smaller cousins, namely exosomes, as it has become apparent that they have the ability to transmit signals including protein and miRNA between cells (Valadi et al., 2007). It therefore seems likely that exosomes are involved in normal physiological, and potentially also pathological processes (Lawson et al., 2016). Since blood contains enormous numbers of exosomes released by platelets, endothelium, and other cell types, it is clearly of interest to investigate their role in the cardiovascular system, and to determine their contribution to cardiovascular diseases (Sluijter et al., 2017, Lawson et al., 2016).

Cardiovascular disease is the major cause of death not only in developed countries but world-wide (Hausenloy and Yellon, 2013). A major cause of cardiovascular disease is damage to the vasculature resulting in gradual build-up of atherosclerotic plaques that can partially occlude the vessel. If this occurs in the heart, it can result in the distal myocardium becoming ischaemic under situations of increased cardiac demand. In some circumstances, the plaque can rupture, exposing the intravascular wall to the coaggulative components of the blood which rapidly cause thrombotic occlusion of the artery, followed by ischaemia, with the development of an acute myocardial infarction. If the clot is not quickly removed by thrombolysis or percutaneous coronary intervention, or is not bypassed surgically (CABG), then the ischaemic myocardium will die. Thus, it is crucial to reperfuse the vessel as quickly as possible. However, it has been known for many years that reperfusion causes a degree of additional injury, referred to as “reperfusion injury” (Hausenloy and Yellon, 2013, Bell et al., 2016, Hausenloy et al., 2017, Lecour et al., 2014). As the final infarct size in patients with acute myocardial infarction (AMI) predicts long-term clinical outcome (Lonborg et al., 2013), it is envisaged that the identification of means of minimizing this injury will reduce patient mortality and morbidity.

To this end, many interventions have been investigated with the aim of reducing reperfusion injury. However, despite their success in animal studies, few have shown efficacy in patients (Bell et al., 2016, Hausenloy et al., 2017, Lecour et al., 2014, Kloner et al., 2017). One of the most potent methods of protecting the heart in experimental models is to subject it to several brief (3–5 min) periods of ischaemia prior to a longer period of injurious ischaemia and reperfusion, a procedure called ischaemic preconditioning (IPC) (Hausenloy and Yellon, 2013, Hausenloy et al., 2017, Lecour et al., 2014). Although the efficacy of IPC has been demonstrated in humans, it is clearly impractical to apply prior to myocardial infarction in the majority of cases that occur spontaneously (Yellon et al., 1993). There is therefore great interest in the potential for remote IPC (RIPC), in which the preconditioning stimulus can be easily applied to a limb remote from the heart, up to the time that the myocardium is reperfused (Hausenloy and Yellon, 2013, Bell et al., 2016, Davidson et al., 2013, Sharma et al., 2015). RIPC is highly effective and reproducible in animal studies (Bromage et al., 2017), and numerous phase I clinical trials have suggested efficacy at preventing myocardial injury in the setting of an AMI (Heusch and Rassaf, 2016), yet it remains to be validated in a larger clinical trial (Hausenloy et al., 2015).

**Fig. 1:**
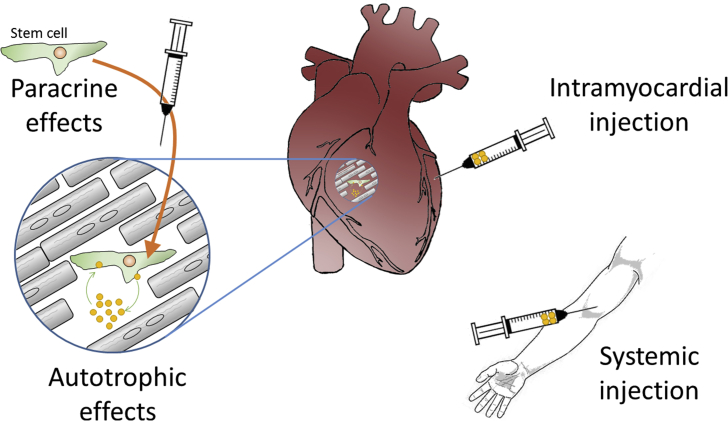
Exosomes can potentially exert effects on the heart via multiple different pathways. 1) Exosomes produced from intramyocardially injected stem cells can exert paracrine effects. 2) Resident cardiac stem cells may cause autotrophic stimulation of themselves or other cell types in the heart. 3) Exosomes injected intramyocardially can affect different cell types directly. 4) Exosomes administered systemically interact with cells of the cardiovascular system, including endothelium, blood cells and the heart.

One alternative approach to protecting the heart against the initial insult of IR injury, is to attempt to restore the lost myocardium, or at least improve its function. Unfortunately, since mammalian cardiomyocytes are terminally differentiated there is extremely limited scope for the spontaneous recovery of myocardium by cardiomyocyte proliferation. However, a great deal of effort has been expended in attempting to regenerate myocardium by injecting different types of stem cells (Madonna et al., 2016). Unfortunately this approach has also been largely unsuccessful in creating new myocardium, despite some encouraging effects being observed with respect to the preservation of existing myocardium, and improvements in cardiac contractile function (Madonna et al., 2016). Since these benefits were clearly not mediated by an increase in cardiomyocyte number, attention has recently turned to factors that may be released from the remaining stem cells, and that may mediate this paracrine effect. In particular, exosomes have been proposed as an important potential paracrine factor (Fig. 1) (Davidson et al., 2017a, Yellon and Davidson, 2014).

**Fig. 2:**
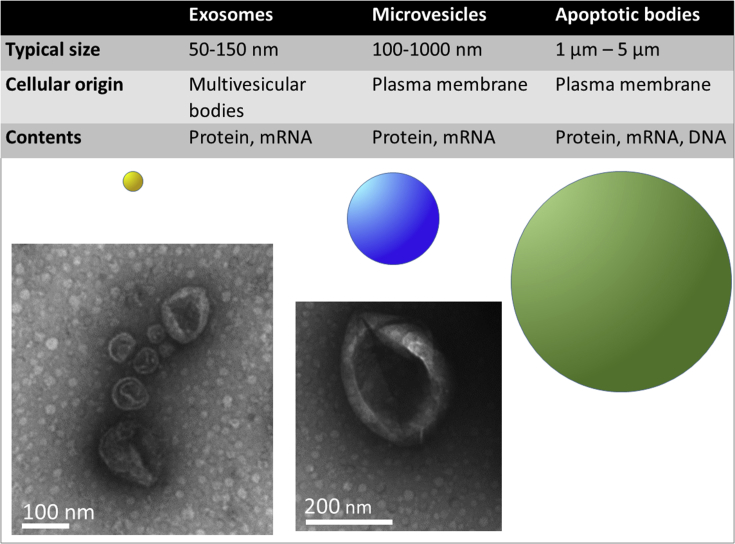
The major characteristics of the different classes of small EV. Representative vesicles are shown to indicate the relative sizes, and transmission electron micrographs demonstrate the “cup-shaped” appearance of the vesicles after preparation. Size bars as indicated.

Exosomes, like all EVs, are enclosed by a lipid bilayer membrane, and contain components originating from their cell of origin. Exosomes are some of the smallest EVs, typically only 50–150 nm in diameter (Fig. 2). This renders them difficult to visualize using standard light imaging techniques, since they are smaller than the wavelength of light. Most isolation procedures are able to achieve a relative concentration of exosomes, but these preparations usually also contain a certain number of MVs and other EVs, due to their overlapping size distributions (Fig. 2). In particular, exosomes’ similar size and/or density to different lipoprotein particles makes them extremely challenging to isolate at high purity from blood. To avoid interference from bovine exosomes, cultured cells may be grown in nominally exosome-free medium, although even here the risk of contamination by lipoprotein particles remains (Shelke et al., 2014). Alternatively, in some studies, cells are cultured for several days in serum-free medium, although this also runs the risk of artefactually altering the cells. Consequently, using current techniques, it is challenging to ascribe particular functional effects specifically to exosomes. For this reason, some studies refer to “exosomal” preparations more conservatively as “small EVs” (sEVs). Given this uncertainty, it is crucial to consider the method of EV isolation used in each individual study, since this determines to a large extent the type of EVs in the preparation. This issue, and the effect of different isolation methods has been extensively reviewed (Sluijter et al., 2017), and is also discussed in other articles in this series.

Despite the above caveats, important clues have been obtained to suggest that EVs do play a role in cardiovascular health and disease (Lawson et al., 2016). Recent reviews have given an excellent description of the role of EVs more broadly in cardiovascular and metabolic disease (Boulanger et al., 2017, Lawson et al., 2016, Sluijter et al., 2014). The aim of the present review is to critically review the literature investigating exosomes, their ability to protect the heart and restore its function after IR injury.

## 2 Mesenchymal stem cell-derived exosomes

Mesenchymal stem cells (MSCs) have generated extensive interest due to their multi-lineage differentiation potential and their ability to be expanded *in vitro* (Madonna et al., 2016, Zomer et al., 2015a). A further advantageous characteristic is that MSCs can be obtained from different tissues, whether foetal, young or adult, although it appears that those derived from younger source tissues are the more potent (Madonna et al., 2016). Interestingly, MSCs also appear to have immunosuppressive properties (De Miguel et al., 2012), raising the possibility that EVs originating from MSCs might be able to confer multiple benefits, not just on the heart directly but via effects on cells of the immune system.

Lai et al. were the first to show that exosomes from MSCs are cardioprotective acutely (Lai et al., 2010a). They used HPLC to purify exosomes released by MSCs in culture and injected them into the tail veins of mice undergoing 30 min myocardial ischaemia via ligation of the coronary artery (Lai et al., 2010a). In injected mice, infarct size was significantly reduced 24 h later (Lai et al., 2010a), and cardiac function was improved at 28 days (Arslan et al., 2013). Importantly, a dose-response study was performed and at least 4 μg/kg exosomes were required to observe significant benefit (Arslan et al., 2013). Furthermore, the MSC exosomes also protected the isolated, perfused heart, meaning that protection was independent of circulating immune cells (Arslan et al., 2013). In the hearts of treated animals, higher levels of ATP and NADH as well as lower levels of oxidative stress were observed but it is difficult to know whether this is a primary effect or is secondary to the smaller infarcts (Arslan et al., 2013). To obtain insight into the potential mechanism of protection, the proteome of MSC exosomes was studied and 730 proteins were identified (Kim et al., 2012) including the proteasome complex responsible for degradation of proteins in the cytosol (Lai et al., 2012). Since the proteasome complex is quite large 30 nm × 12 nm there is a risk that it can co-elute with the smaller exosomes. Interestingly, however, exosomes have long been known to contain polyubiquitinated proteins, suggesting they share a relationship with protein degradation pathways (Eitan et al., 2016). The source of MSC cells used in the above studies is somewhat unusual, in that they were derived from human ES cells by several rounds of *in vitro* passaging and selection (Lai et al., 2010a). However, the origin of the MSCs does not appear to affect their ability to protect the heart. MSCs isolated from human foetal limb, kidney and liver tissue were all equally effective at producing exosomes that were protective in an *in vivo* mouse model of myocardial IR injury (Lai et al., 2010b).

MSC exosomes have also been shown to be protective in a model of permanent coronary artery ligation, in which the myocardium is subject to continuing ischaemia without reperfusion. In these experiments, exosomes were isolated from human umbilical cord MSCs by ultracentrifugation then intravenously administrated immediately following ligation of the left anterior descending (LAD) coronary artery in rats (Zhao et al., 2015). Four weeks later, improvements in cardiac systolic function were noted as well as a reduction in cardiac apoptosis and fibrosis (Zhao et al., 2015). The MSC exosomes also promoted tube formation and migration of an endothelial cell line *in vitro* (Zhao et al., 2015). However, it must be noted that the exosomes isolated in this study were atypically small, measuring only 20–80 nm by nanoparticle tracking analysis (NTA), and their appearance by transmission electron microscopy (TEM) is more reminiscent of spherical lipoprotein particles than the typical “cup-shaped” collapsed vesicular structure of true exosomes (Zhao et al., 2015). This may reflect the fact that the MSCs were cultured using medium containing serum, which contains high concentrations of lipoprotein particles that overlap with exosome in diameter and readily co-purify with exosomes (Shelke et al., 2014).

**Fig. 3:**
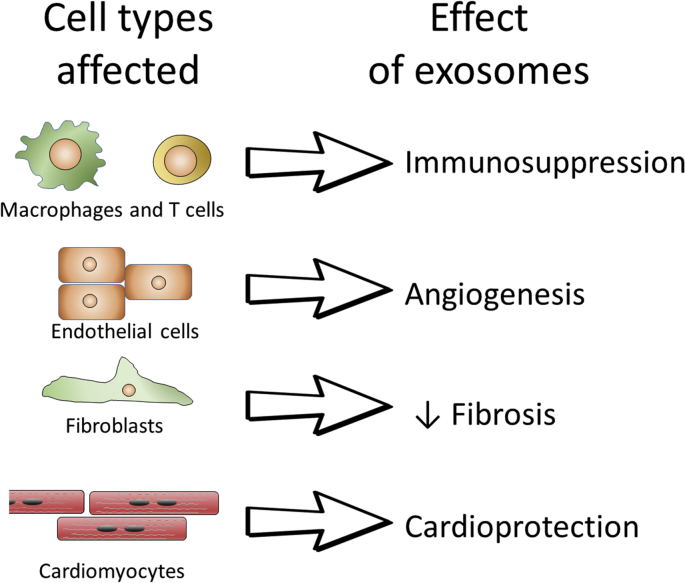
Some of the major effects that have been reported of exosomes that are relevant to the ischaemic heart, and the cell types that have been reported to be involved in the effect. See text for details.

In addition to an acute effect in reducing infarct size noted above, part of the improvement in long-term cardiac function could be due to effects on cardiac remodelling and revascularization (Fig. 3). Indeed, the parent MSCs are known to promote angiogenesis via the secretion of numerous proteins that might influence endothelial growth and vessel sprouting, including VEGF and Hepatocyte Growth Factor (HGF) (Zomer et al., 2015a). However, there is evidence that exosomes and larger EVs also mediate part of this proangiogenic mechanism (Todorova et al., 2017). For example, Gong et al. isolated exosomes isolated from a cell line of MSCs called C3H10T1/2 using Exoquick and found that they stimulated endothelial proliferation *in vitro* (Gong et al., 2017). Shabbir et al. isolated exosomes from human bone-marrow-derived-MSCs (BM-MSCs) using differential ultracentrifugation, after first clearing the calf serum used to culture the cells of exosomes (Shabbir et al., 2015). The BM-MSC exosomes stimulated tube formation of endothelial cells *in vitro*, as well as the proliferation and migration of fibroblasts (Shabbir et al., 2015). Liang et al. isolated exosomes by filtration and ultracentrifugation from cultured human adipose-derived MSCs (AD-MSCs) in serum-containing medium, and showed that they stimulated endothelial tube formation both *in vitro* and in a Matrigel plug *in vivo* (Liang et al., 2016). Finally, Hu et al. showed that MSC exosomes were able to enhance microvessel density and blood perfusion in an *in vivo* mouse model of hind limb ischaemia. The exosomes were isolated from iPSC-derived MSCs by a process of ultrafiltration and density gradient purification, and were further verified to stimulate endothelial migration, proliferation, and tube formation *in vitro* (Hu et al., 2015).

In attempting to determine the mechanism by which MSC exosomes stimulate angiogenesis, many studies have focussed on their miRNA content. Liang et al. determined that miR-125a was enriched in their AD-MSC exosomes, and proposed that by transferring this miRNA to endothelial cells they promote angiogenesis by repressing DLL4 (Liang et al., 2016). Gong et al. detected multiple pro-angiomiRs in their MSC-exosomes, and by using loss and gain of function experiments found that miR-30b was important for the stimulation of endothelial proliferation *in vitro* (Gong et al., 2017). Integrated transcriptomic and proteomic analysis of the molecular cargo of EVs from porcine AD-MSCs demonstrated that their miRNA and mRNA cargo targeted and encoded transcription factors (TFs), and the EVs were enriched for proteins that support extracellular matrix remodelling, blood coagulation, inflammation, and angiogenesis (Eirin et al., 2017). A possible caveat with this study is that cells were reportedly cultured in medium containing platelet lysate (Eirin et al., 2017), and platelets are known to be major sources of EVs including both MVs and exosomes (Heijnen et al., 1999). Overall, it seems the particular balance of pro-angiogenic miRNAs that stimulate angiogenesis may depend on the source of MSCs, and is quite likely to vary with the experimental model. Analysis of miRNA in exosomes released by cultured cells can be challenging, not only because of the low yield, but because of potential interference from miRNA contained in serum or other additives added to the cell culture (Tosar et al., 2017). If, on the other hand, serum is omitted, there may be quite severe effects on the growth and health of the cells.

An additional possible benefit of MSC exosomes on cardiac function could be via direct effects on cardiac contractility. For example, exposure of human engineered cardiac tissue to exosomes from human MSCs increased expression of calcium-handling genes such as SERCA2a and the L-type calcium channel, and consequently improved contractility while protecting from potential pro-arrhythmic effects of heterocellular coupling in the engineered tissue (Mayourian et al., 2017). Uptake of fluorescently labelled exosomes was shown using cardiomyocytes differentiated *in vitro* from stem cells. Although suggestive of an effect on cardiomyocytes, such stem cell-derived cardiomyocytes are at a relatively immature stage, therefore these experiments do not provide direct evidence that MSC exosomes can affect the contractility of mature cardiomyocytes in the adult heart.

Attempts have been made to genetically engineer MSC exosomes so that they deliver even greater cardiovascular benefits. MSCs overexpressing GATA-4 produced exosomes that were able to reduce infarct size and improved cardiac function when injected intramyocardially prior to coronary artery ligation (Yu et al., 2014). Their enhanced ability to protect the heart was attributed to their increased content of anti-apoptotic miRs, in particular miR-19a which resulted in increased activity of the PI3K/Akt kinase pathway (Yu et al., 2014). This pathway is well-established as being central to the so-called “Reperfusion injury Salvage Kinase (RISK)” cardioprotective signalling pathway (Hausenloy and Yellon, 2013, Hausenloy et al., 2017). Exosomal uptake into primary rat neonatal cardiomyocytes was visualized *in vitro*. Kang et al. overexpressed CXCR4 in MSC cells, since this receptor for SDF-1α is important in homing and targeting of stem cells (Bromage et al., 2014, Ziff et al., 2017). Furthermore, activation of the CXCR4-SDF-1α pathway can protect the heart from IR injury, and may be important for the suppression of heart failure (Sluijter et al., 2017, Davidson et al., 2013, Bromage et al., 2014, Ziff et al., 2017, Malik et al., 2015). Exosomes released by MSC-CXCR4 cells were found to significantly upregulate levels of IGF-1α and pAkt in cardiomyocytes, as well as enhancing VEGF expression and vessel formation *in vitro* (Kang et al., 2015). Ma et al. engineered human umbilical cord MSCs to overexpress Akt and found that the exosomes produced by these cells had a greater ability to stimulate angiogenesis (Ma et al., 2017). After intravenous injection, cardiac function was improved following coronary artery ligation in rats (Ma et al., 2017). Interestingly, Akt itself was detected in these exosomes, although the pro-angiogenic stimulation was attributed to platelet-derived growth factor D (PDGF-D) that was also present within them (Ma et al., 2017).

As mentioned, another characteristic of MSCs is their immunosuppression (Fig. 3). It is therefore important to establish whether MSC-derived exosomes have the same intriguing capacity. A recent well-performed study used size-exclusion chromatography to obtain highly purified EVs from cultured umbilical cord MSCs (Monguio-Tortajada et al., 2017). Cryo electron microscopy and protein markers clearly demonstrated the presence of exosomes without protein contamination, although a size estimate of ∼170 nm by NTA suggests there may have also been larger vesicles present. Interestingly, in the different fractions analysed, only the EVs were able to greatly immunosuppress polyclonal T cell activation (Monguio-Tortajada et al., 2017). The EVs did not have any effect on macrophage polarization or cytokine secretion (Monguio-Tortajada et al., 2017). As the authors note, this illustrates the importance of working with well purified EV preparations, since the non-EV fraction induced the expression of CD163 and CD206 and some production of TNF-α by monocytes (Monguio-Tortajada et al., 2017). Intriguingly, one of the means by which MSCs regulate immunomodulation involves the release of the lipid based signalling molecule prostaglandin E2 (Prockop and Oh, 2012). As lipids, it is perhaps not surprising that prostaglandins have been found to be associated with the lipid membrane of exosomes (Subra et al., 2010). However, whether PGE2 is involved in the mechanism of immunosuppression by MSC exosomes has not yet been investigated.

Although the results described in this section suggest the exciting possibility of being able to harvest exosomes from cultured MSCs and obtaining most if not all of their benefits in the absence of the potential risks associated with the injection of live cells, excitement must be tempered by several important caveats. Firstly, despite clear evidence for uptake of exosomes into immature cardiomyocytes and cell lines, the extent to which exosomes are able to enter and deliver contents such as miRNA to mature, adult cardiomyocytes is unclear. Only a few studies have investigated this, and have concluded that, actually, there is relatively little uptake of exosomes into primary cardiomyocytes (Vicencio et al., 2015, Agarwal et al., 2017, Gray et al., 2015). The evidence for uptake into cardiomyocytes *in vivo* is even more limited, being mostly limited to the indirect demonstration of altered gene expression, which may be attributable to the delivery of miRNAs, but could equally be a secondary response to receptor activation by exosomal-surface proteins. One alternative is that the exosomes act on the endothelium, as these expert scavenging cells can certainly take up all types of exosomes including MSC exosomes (Gong et al., 2017, Shabbir et al., 2015, Liang et al., 2016, Hu et al., 2015). Furthermore, in some experiments the exosomes may not even be acting directly on the myocardium, but may be reprogramming macrophages or monocytes that have engulfed them. This hypothesis is being actively investigated as the potential method of action of CPC exosomes (de Couto et al., 2015, de Couto et al., 2017, Kanazawa et al., 2016).

A surprising but consistent finding in stem cells studies is that cardiac retention and engraftment of intravenously administered MSCs is very low. This has been interpreted as indicating that early paracrine effects may confer prolonged benefit, perhaps through phenotype alteration of local macrophages for example. Alternatively, they may mediate systemic anti-inflammatory effects. For example, Luger et al. found that, despite widespread distribution to tissues aside from the heart, intravenously-administered MSC treatment after acute MI ameliorated late left ventricular remodelling, apparently via systemic anti-inflammatory activities (Luger et al., 2017). Furthermore, if this is the case, it may not be necessary for any MSCs to engraft in the heart at all. In one intriguing study, MSCs were injected subcutaneously in mice after MI such that they did not migrate detectably from the injection site, and LV function was improved (Preda et al., 2014). The protein pentraxin 3 was suggested to be involved in the mechanism of this remote type of protection (Preda et al., 2014). The potential role of exosomes in these observations has not yet been investigated.

## 3 Cardiac stem cell-derived exosomes

**Fig. 4:**
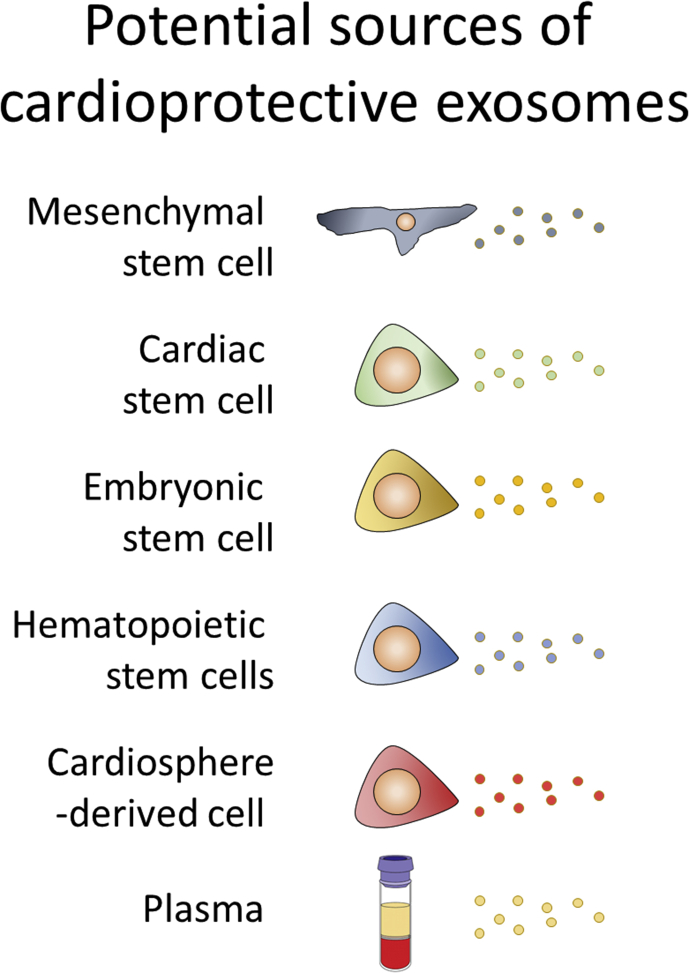
The different potential sources of exosomes discussed in this review. Although each type of cell has certain unique characteristics, the exosomes they produce are notable for the consistent array of effects they induce.

MSCs are not the only cell type being investigated in terms of cardiac cell therapy (Fig. 4). There is great interest in the potential for various cardiac resident stem cells, including c-kit^+^ cells, WT1^+^ cells and W8B2^+^ cells to name a few, to restore function to the injured heart, although there is also a great deal of debate as to which of these represent bone fide, cardiac-resident stem cells (Zhang et al., 2015).

Human Sca1^+^ cardiac progenitor cells (CPCs) harvested from aborted foetuses have been shown to secrete exosomes into culture medium (Vrijsen et al., 2010). After purification by ultracentrifugation and density gradient, these exosomes can stimulate the migration of endothelial cells in an *in vitro* scratch wound assay (Vrijsen et al., 2010). In these experiments, migration was blocked by a neutralizing antibody against extracellular matrix metalloproteinase inducer (EMMPRIN) (Vrijsen et al., 2010). CPCs can also be obtained from the right atrial appendages of patients undergoing cardiac surgery. Exosomes purified from these cultured are also capable of stimulating endothelial tube formation (Barile et al., 2014). Furthermore, when injected into the infarct border zone these CPCs-EVs reduced cardiomyocyte apoptosis and scar, increased both viable mass in the infarct area and blood vessel density, and prevented the early impairment of ventricular function at day 7 in a permanent ligation rat model of acute MI (Barile et al., 2014). Interestingly, in this study, EVs isolated from dermal fibroblasts did not confer any benefit, suggesting the benefits are specific to stem cells (Barile et al., 2014). It is not entirely clear what characteristics of stem cells are important in order for them to produce exosomes that are beneficial to the ischaemic heart. Indeed, it has previously been suggested that *ex vivo* cultured fibroblasts and MSC are, in fact, indistinguishable by all commonly used parameters (Hematti, 2012). Both fibroblasts and MSCs are multipotent. Both have immunosuppressive qualities (Haniffa et al., 2009). A comparison of the proteome and miRnome of exosomes from stem cells and fibroblasts may reveal important differences.

An important factor affecting the potency of CPCs may be the age of the donor. In a study comparing human c-kit^+^ CPCs from adults and neonates, those from neonates proliferated more *in vitro* and led to greater myocardial recovery in rats when injected intramyocardially after permanent coronary artery ligation (Sharma et al., 2017). Interestingly, exosomes produced by the neonatal CPCs appeared to be responsible for a large proportion of this benefit, although a non-exosomal fraction also delivered some benefit (Sharma et al., 2017). Exosomes from hypoxic (but not normoxic) c-kit^+^ rat CPCs were found to improve cardiac function and reduce fibrosis after ischaemia followed by 3 weeks reperfusion (Gray et al., 2015). They were also found to enhanced tube formation of endothelial cells and decreased profibrotic gene expression in TGF-β-stimulated fibroblasts *in vitro* (Gray et al., 2015). A separate study found that c-kit^+^ CPC exosomes isolated from the right atrial appendages of children undergoing cardiac surgery were not protective when administered intramyocardially to athymic rats after IR, in comparison to those from neonatal hearts (Agarwal et al., 2017). Interestingly, hypoxic culture could restore some of the protective ability of CPCs exosomes (Agarwal et al., 2017).

When c-kit^+^ stem cells are isolated from the heart it is important to exclude mast cells, which also express c-kit^+^, but can be excluded on the basis of being CD45^+^. This step is particularly important because exosomes are also released by mast cells (Eldh et al., 2010). Interestingly, exposure of mouse mast cells to oxidative stress causes an alteration in the mRNA content of exosomes released (Eldh et al., 2010). Furthermore, these exosomes can influence the response of other cells to oxidative stress by providing recipient cells with a resistance against oxidative stress (Eldh et al., 2010).

Based on the concept that exosomes may mediate part of the paracrine benefit of transplanted stem cells, MSC exosomes have been used to “precondition” stem cells before transplantation. In these experiments, cardiac stem cells (CSCs) were exposed *in vitro* to exosomes isolated from cultured rat BM-MSCs using Exoquick-TCTM (Zhang et al., 2016). This was found to stimulate the proliferation, migration, and angiotube formation of the CSCs (Zhang et al., 2016). When injected in a rat myocardial infarction model, the preconditioned CSCs had significantly improved survival, enhanced capillary density, reduced cardiac fibrosis, and restored long-term cardiac function (Zhang et al., 2016). However, since the isolated particles measured only 10–80 nm there is a concern that lipoprotein particles from the serum may have contributed to the preconditioning effects (Zhang et al., 2016).

Ong et al. used a novel genetic strategy to try to enhance the healing properties of Sca1^+^ murine CPCs by targeting the supporting stromal cells. By co-delivering a minicircle plasmid encoding HIF-1 with the CPCs, the surrounding stromal cells were made to overexpress HIF-1, which appears to have increased transfer of miR-126 and miR-210 via exosomes to the recipient CPCs, activating prosurvival kinases and inducing a glycolytic switch (Ong et al., 2014).

Recently, a novel population of cardiac resident stem cells were described, which were obtained from adult human atrial appendages and shown to be positive for the W8B2 antigen TNAP (Tissue non-specific alkaline phosphatase), that is selectively expressed on MSCs (Zhang et al., 2015). W8B2^+^ CPCs express mesenchymal markers and can differentiate into cardiovascular lineages. The conditioned medium had prosurvival, proangiogenic, and pro-migratory effects on endothelial cells, as well as protective cells on neonatal rat cardiomyocytes. Intramyocardial transplantation of human W8B2^+^ CPCs markedly improved cardiac function of rats 1 week after myocardial infarction. It was recently demonstrated that W8B2^+^ cells secrete exosomes that may mediate these effects (Nie et al., 2017).

## 4 Embryonic stem (ES) cells-derived exosomes

Extrapolating from the advantages described above with using CPC exosomes from younger donors, it might be hypothesized that exosomes produces by embryonic stem (ES) cells, which are by definition even younger, and which furthermore are totipotent as opposed to multipotent, might be even more efficacious in protecting and regenerating the heart. As with studies using other sources of exosomes, exosomes from mouse ES cells enhanced neovascularization, cardiomyocyte survival and reduced fibrosis post infarction (Khan et al., 2015). Interestingly, after 8 weeks, an increase in c-kit^+^ CPCs and new cardiomyocytes was observed in the heart of mice receiving these exosomes (Khan et al., 2015). Furthermore, it might be speculated that these new CPCs would contribute their own beneficial exosomes to the healing process in a kind of “virtuous circle”. Interestingly, miR-294 was found to be particularly enriched in exosomes from ES cells, and delivery of this miRNA to CPCs was sufficient to promote their increased survival, cell cycle progression and proliferation (Khan et al., 2015).

Human ES cell-derived cardiovascular progenitors (hESC-Pg) have been administered intramyocardially 2–3 weeks after coronary artery occlusion, and were found to enhance recovery of cardiac function despite no remaining cells being detected in the heart (Kervadec et al., 2016). It was hypothesized that EVs mediated the benefits of transplanted cells, since they were able to recapitulate the effects (Kervadec et al., 2016). Although exosomes were present in the isolated EVs, the effect could not be reliably assigned to them, since there were also a large number of MVs present (Kervadec et al., 2016).

## 5 Hematopoietic stem cell-derived exosomes

Bone marrow derived CD34^+^ hematopoietic stem cells have been investigated as a means of promoting angiogenesis in ischaemic hearts, thereby preserving cardiac function. Exosomes purified from human CD34^+^ stem cells by ultracentrifugation through a sucrose gradient cushion were found to increase endothelial cell viability, proliferation, and tube formation (Sahoo et al., 2011). Interestingly, although exosomes from CD34^+^ cells stimulated angiogenesis in Matrigel plugs implanted *in vivo*, those from the mononuclear cells remaining after depletion of CD34^+^ cells did not (Sahoo et al., 2011). CD34^+^ exosomes were therefore proposed to mediate the proangiogenic paracrine activity of the parent cells (Sahoo et al., 2011). Human CD34^+^ stem cell exosomes were also found to induce repair of ischaemic hind limb by stimulating angiogenic mechanisms via a mechanism involving transfer of miR-126-3p (Mathiyalagan et al., 2017). In these experiments, the greatest internalization of CD34^+^ exosomes was observed into endothelial cells relative to smooth muscle cells and fibroblasts of ischaemic hindlimbs (Mathiyalagan et al., 2017).

When injected into the ischaemic border zone of immune-compromised mice after coronary artery ligation, human CD34^+^ cells were found not to be protective (Mackie et al., 2012). However, if they were first engineered to overexpress the known angiogenic factor, sonic hedgehog (Shh), ventricular size and cardiac function were maintained (Mackie et al., 2012). The modified cells were shown to release Shh in exosomes and activate the canonical Shh signalling pathway in recipient cells. Interestingly, microvesicles generated from activated/apoptotic human T lymphocytes have also been found to contain Shh naturally, and when administered to mice receiving angiotensin II, they were found to revert hypertension and endothelial dysfunction through a mechanism associated with Shh-induced production of nitric oxide, and by decreasing levels of oxidative stress (Marrachelli et al., 2013).

## 6 Exosome from cardiosphere-derived cells

Cardiosphere-derived cells (CDCs) are a unique type of cardiac-derived stem cell originally identified in the Marbán lab that are expanded *ex vivo* from patient cardiac biopsies (Smith et al., 2007). CDCs exhibit multi-lineage differentiation and have been shown in various preclinical models to improve cardiac function after delivery to ischaemic myocardium (Cheng et al., 2014a, White et al., 2013). CDCs contain a mix of cell types, including some c-kit^+^ cells, but the majority of the “active” cell type are believed to be CD105^+^/CD90^−^/c-kit^−^ cells (Cheng et al., 2014b). Of particular interest, a phenomenon coined “cellular postconditioning” has been identified whereby CDCs have been shown to protect the hearts of pigs or hypertensive rats when administered 20–30 min after the onset of reperfusion. This characteristic of delayed protection is somewhat of a “holy grail” for cardioprotection, since it implies that procedures can be implemented after a substantial delay and still provide significant patient benefit. The mechanism of this delayed protection appears to be due to their effect on polarizing cardiac macrophages toward a distinctive cardioprotective phenotype (de Couto et al., 2015, Kanazawa et al., 2015, Kanazawa et al., 2016). However, in this regard, it is interesting to note that similar benefits have been obtained using intravenous administration of artificial liposomes presenting phosphatidylserine on their surface, which causes them to be taken up by macrophages, improving the resolution of inflammation, stimulating angiogenesis and elicit infarct repair (Harel-Adar et al., 2011).

After a promising, albeit small, phase I trial, the ALLSTAR multicentre randomized, double-blind, placebo-controlled trial of allogeneic CDCs in patients with myocardial infarction (MI) and ischemic left ventricular dysfunction progressed to a larger phase 1/2 safety and efficacy trial of intracoronary delivery (Chakravarty et al., 2017). Unfortunately, the trial was recently reported to have been terminated early due to low probability of attaining its primary endpoint of a reduction in myocardial scarring due to infarction (Capricor Therapeutics, 2017). However, given that the mechanism of action of CDCs in experimental models seems to be via the release of paracrine factors, there remains great interest in the potential for CDC exosomes to confer a cardioprotective benefit.

Human CDC exosomes have been administered to pigs in both acute and chronic models of cardiac ischaemia. Interestingly, they reduced infarct size and preserved systolic function after intramyocardial but not intracoronary delivery (Gallet et al., 2017). Notably, significantly more allo-antibodies were observed in hearts that had received exosomes, indicative of a mild immune response. It is also worthwhile to consider that the exosomes used in these studies were prepared by precipitation with polyethylene glycol (PEG), which is generally not recommended as the exosomes are regarded as being relatively impure (Paolini et al., 2016). Furthermore, the effect of the residual PEG on cardiac function is not known. Interestingly, as had been observed with CDC cells themselves, administration of CDC exosomes 20–30 min after reperfusion reduced infarct size in both rat and pig models of MI measured 48 h later (de Couto et al., 2017).

In these studies, the cardioprotective effects were said to derive from exosomal transfer of miR-181b from CDCs into macrophages which reduced PKCδ transcript levels (de Couto et al., 2017). Interestingly, although fibroblast exosomes were not protective in this model, those that had been loaded selectively with miR-181b were then able to alter macrophage phenotype and deliver cardioprotection (de Couto et al., 2017).

Part of the longer-term benefit of CDCs may also be via reprogramming of resident fibroblasts. When dermal fibroblasts were primed with exosomes from rat CDCs for 24 h *in vitro*, they were rendered therapeutic, and were able to stimulate angiogenesis and improve cardiac remodelling when injected intramyocardially 1 month after induction of MI in rats (Tseliou et al., 2015). Interestingly, the primed fibroblasts secreted higher levels of stromal-cell-derived factor 1 (SDF-1α) and vascular endothelial growth factor (VEGF) (Tseliou et al., 2015).

Curiously, the acute reduction in infarct size mediated by CDC exosomes has been attributed to a Y RNA fragment they contain called EV-YF1, with the potency of CDCs *in vivo* correlated with the content of EV-YF1 (Cambier et al., 2017). Y RNAs are small non-coding RNAs that form components of the Ro60 ribonucleoprotein particle involved in DNA replication. Exosomes transfer EV-YF1 from CDCs to macrophages, resulting in them increasing expression of the immunosuppressive cytokine Il-10 (Cambier et al., 2017). CDC exosomes also contain more commonly known small RNAs including miRNAs, particularly miR-146a, which appears to mediate part of the regenerative and functional effects of CDC exosomes injected into mice (Ibrahim et al., 2014).

## 7 Plasma exosomes

Exosomes and other EVs are present at high concentrations in blood, and may have a role in communication between organs or between cells within tissues (Davidson et al., 2017a). Plasma exosomes isolated from human or rat blood by differential ultracentrifugation have been shown to be cardioprotective, reducing infarct size when administered intravenously to rats, or added to isolated, perfused rats hearts or primary cardiomyocytes (Vicencio et al., 2015). HSP70 present on the surface of plasma exosomes was shown to stimulate the TLR4 receptor on cardiomyocytes, leading to activation of the ERK1/2 pathway, which is a central component of the cardioprotective RISK pathway (Vicencio et al., 2015). Inhibition of any of these steps also blocked cardioprotection (Vicencio et al., 2015). However, it was recently demonstrated that exosomes isolated using the same technique from the blood of rats or humans with type II diabetes were unable to activate ERK1/2 and had lost this cardioprotective ability (Davidson et al., 2017b). These data suggest that plasma exosomes might exert a continuous, mild stimulatory effect of cardioprotective pathways in the heart. Furthermore, it has been suggested an increase in the number of activity of plasma exosomes might be responsible for the transmission of a cardioprotective signal from the remotely conditioned limb to the heart during the process of RIPC (Yellon and Davidson, 2014). Indeed, in both humans and rats, RIPC has been found to increase the quantity of exosomes in the blood (Vicencio et al., 2015). However, only a marginal, non-significant increase in protection was observed with exosomes isolated after RIPC, suggesting this is not the only mechanism involved in this phenomenon (Davidson et al., 2013, Vicencio et al., 2015). On the other hand, evidence obtained using experiments in which the effluent from one perfused heart is transferred to a second heart suggests that exosomes may be able to transmit protection between organs (Giricz et al., 2014). Probably, the definitive answer regarding the requirement for exosomes in RIPC awaits the development of a specific inhibitor of *in vivo* exosomes production, as discussed in section 8.

It may turn out to be the case that EVs other than exosomes mediate RIPC, though here the data is similarly contrasting. Jeanneteau et al. found that RIPC caused an increase in the number of circulating endothelial MVs and procoagulant MVs in rats and humans, but infarct size was not altered by intravenous injection of MVs from an RIPC-treated rat (Jeanneteau et al., 2012). However, Ma et al. found that MV injection reduced infarct size (Ma et al., 2015), perhaps because they combined the MVs isolated from 3 rats to inject into each rat, leading to a greater overall increase in EV number (Ma et al., 2015).

## 8 Perspectives

Despite the numerous promising studies the have observed impressive cardioprotective benefits with exosomes administered to hearts, either after acute IR, or in a chronic model of permanent ischaemia, the results must be interpreted with caution. Many studies used relatively impure populations of vesicles. Some precipitation methods of purification such as Exoquick can result in relatively low purity (Sluijter et al., 2017, Paolini et al., 2016). Unfortunately, a practical method of isolating high yields of very pure exosomes remains to be developed. Lipoprotein particles and/or proteins remain a major potential contaminant from serum-containing medium using any commonly used technique including differential centrifugation, precipitation, and size-exclusion chromatography (Sodar et al., 2016, Yuana et al., 2014). In the meantime, the most pragmatic option seems to be to isolate exosomes from cells cultures for 1–2 days in serum-free medium, although it is essential to verify that the cells have not started to undergo apoptosis. In any case, this period of serum starvation is known to dramatically alter the metabolism of cultured cells, and the exosomes that are produced under this condition may be quite distinct from those that are produced in serum or *in vivo*.

Several techniques are commonly used to verify the presence of exosomes in an isolate, but all are subject to limitations. TEM is highly selective and in any case, many (if not most) figures of “exosomes” presented in papers bear little resemblance to the classical “cup-shaped” vesicle expected when the exosomes collapse during drying in preparation for TEM (Thery et al., 2006). NTA can estimate size distribution and concentration of particles, but cannot distinguish between exosomes, lipoprotein particles, protein aggregates, or other similarly sized particles such as calcium precipitates. Western blotting can demonstrate the presence of exosome “marker proteins” such as Alix, HSP70, CD63, CD81 and CD9, but by itself is not a good indication of purity, although the demonstration of the absence of other membrane proteins such as ER and plasma membrane can help in this regard. Furthermore, some of the aforementioned marker proteins (particularly CD63) seem to be expressed on other small EVs including MVs and so are not very discriminatory for exosomes (Kowal et al., 2016). For all of these reasons, the development of more robust techniques to isolate exosomes and to validate their purity is essential to avoid them becoming the new “snake oil” – able to cure anything, but without anyone really knowing what is in them.

Many experiments demonstrating functional effects with exosomes have been performed *in vitro* using cultured cells (Fig. 4), and although these experiments are informative, the extent to which the effects reflect real *in vivo* processes is not entirely clear. Cleverly designed experiments will be needed, such as those using *in vivo* transfer of Cre mRNA to demonstrate that EVs can transfer metastatic behaviour from cancer cells to benign cells (Zomer et al., 2015b).

Reliable tracing of exosomes remains challenging, particularly *in vivo*. Fluorescent lipophilic dyes are sometimes used for this purpose, but can be misleading since they incorporate avidly into lipoprotein particles (present in serum used for cell culture), and even bind efficiently to protein components of the medium that are unavoidable for the culture of healthy cells (Takov et al., 2017). Thus, it is difficult to interpret experiments using these dyes. Another approach is to radiolabel vesicles. In several studies, intravenously administered exosomes have been observed to accumulate in lung and liver. However, given some of the limitations and caveats discussed above concerning the purity of exosome preparations, it is difficult to be confident that this is not due to impurities. A more robust assay has been developed which involves expression of luciferase and noninvasive bioluminescence imaging (Lai et al., 2014). Using this method, labelled B16-BL6 exosomes were observed to disappear very quickly from the blood circulation with a half-life of only ∼2 min (Takahashi et al., 2013). However, most luminescence was still observed in the liver and lungs. Therefore, it seems that intramyocardial injection may be necessary to achieve sufficient delivery of exosomes to the heart. In this regard, a recent study demonstrated that CDC exosomes were effective in pigs after intramyocardial but not intracoronary delivery post reperfusion (Gallet et al., 2017). Ultimately, new methods must be developed to improve targeted delivery to the heart of exosomes delivered intravenously.

One important consideration is that, despite reproducible benefits of stem cell administration in animal experiments, substantial debate remains as to whether stem cell implantation provides any benefit at all to patients with ischaemic heart disease (Gyongyosi et al., 2016). While it seems certain that cell engraftment is rare, and cardiac regeneration even rarer, the hypothesis that they provide a significant benefit to cardiovascular function via a paracrine effect is not well substantiated by current clinical data. In this case, it is conceptually challenging to understand how the purified paracrine factor (i.e.: exosomes) can be expected to be any more successful than the parent cells. One possibility, which seems quite likely, is that the exosomes produced by *in vitro* cultured cells have different characteristics to those produced by cells *in vivo*. It may therefore be possible to use stem cells as an artificial bio-reactor producing beneficial exosomes *ex vivo*. The first experiments administering exosomes *in vivo* have begun, although the regulations regulating this new biologic remain relatively poorly defined (Lener et al., 2015). One attractive aspect of exosomes is that they may potentially be able to confer some of the benefits of stem cells, without the limitation such as the risk of teratomas developing from transplanted stem cells (Madonna et al., 2016).

Ideally, to prove that exosomes mediate an effect, one would use chemical or genetic suppression of exosome production to investigate whether they also suppress the effect. Unfortunately, current inhibitors such as GW4869 are fairly non-specific, and though Rab proteins are generally thought to be involved in exosome production, the precise member of this large family appears to vary between cell types (Colombo et al., 2014). In the meantime, one must rely on experiments such as those demonstrating that an affect is absent in conditioned medium depleted of exosomes. The majority of functional experiments to date have been performed using cells in culture, and their significance *in vivo* is difficult to ascertain.

Emerging evidence suggests that there are multiple subtypes of small EVs, which may have different characteristics. For instance, it was recently shown that immature and mature dendritic cells release EVs that differ in their capacity to orient T helper (Th) cell responses (Tkach et al., 2017). By affinity isolation of EVs with different contents of cell membrane lipids, MSCs were shown to release at least three different subtypes of EVs, although it is not known if they exhibit different functionalities (Lai et al., 2016). This fact renders it important firstly to ensure isolations of exosomes are as pure as possible, and secondly to investigate possible diverse effects of difference subtypes of EVs or exosomes.

It is also important to be aware that factors influencing the differentiation status of stem cells are likely to influence the functionality of exosomes that are produced. In particular, examples given in the sections above illustrate how the microenvironment or disease conditions such as inflammation, diabetes (hyperglycaemia) and hypoxia can dramatically alter the contents, packaging and functionality of exosomes derived from phenotypically similar stem cells.

In summary, given the uncertainties regarding the purity of exosome isolates, it is difficult to be certain which of the many qualities attributed to exosomes (Fig. 3) is indeed mediated by them, and which are artefacts due to isolation procedures or contaminants. To this end, in order to improve the quality of the data and establish with confidence the potential for exosomes to protect the heart, it is very important to adhere to some of the recently published consensus recommendations on exosome methods (Sluijter et al., 2017, Lener et al., 2015, van der Pol et al., 2016, Witwer et al., 2013, Coumans et al., 2017).

However, several important conclusions can be drawn from data published to date. These include the observation that exosomes work across species, from human to mice and pigs, suggesting a highly conserved mechanism. Furthermore, the beneficial effect of exosomes seems to universally present in exosomes isolated from stem cells of different origin, as well as those from plasma. On the other hand, exosomes from fibroblasts are generally found to be inert. The mechanism of protection is difficult to ascertain, and the results of miRNA studies in particular are not highly consistent. However, generally although exosomes appear to have immunosuppressive effects there have been some reports of a minor immune response after injection (Gallet et al., 2017). Given their long duration of action, the hypothesis that exosomes alter or reprogram endogenous cells of the immune system for example, is attractive. One thing that is certain – despite exosomes having once been thought of as nothing more than cellular debris, there remains a great deal to be discovered about these enigmatic vesicles.
